# Depression, Anxiety, and Lifestyle Among Essential Workers: A Web Survey From Brazil and Spain During the COVID-19 Pandemic

**DOI:** 10.2196/22835

**Published:** 2020-10-30

**Authors:** Raquel Brandini De Boni, Vicent Balanzá-Martínez, Jurema Correa Mota, Taiane De Azevedo Cardoso, Pedro Ballester, Beatriz Atienza-Carbonell, Francisco I Bastos, Flavio Kapczinski

**Affiliations:** 1 Institute of Scientific and Technological Communication and Information in Health Oswaldo Cruz Foundation Rio de Janeiro Brazil; 2 Department of Medicine, University of Valencia CIBERSAM Valencia Spain; 3 Department of Psychiatry and Behavioural Neurosciences McMaster University Hamilton, ON Canada; 4 Neuroscience Graduate Program McMaster University Hamilton, ON Canada; 5 School of Medicine University of Valencia Valencia Spain; 6 Bipolar Disorder Program, Laboratory of Molecular Psychiatry Hospital de Clínicas de Porto Alegre Instituto Nacional de Ciência e Tecnologia Translacional em Medicina Porto Alegre Brazil; 7 Department of Psychiatry Universidade Federal do Rio Grande do Sul Porto Alegre Brazil

**Keywords:** COVID-19, depression, anxiety, lifestyle, Brazil, Spain

## Abstract

**Background:**

Essential workers have been shown to present a higher prevalence of positive screenings for anxiety and depression during the COVID-19 pandemic. Individuals from countries with socioeconomic inequalities may be at increased risk for mental health disorders.

**Objective:**

We aimed to assess the prevalence and predictors of depression, anxiety, and their comorbidity among essential workers in Brazil and Spain during the COVID-19 pandemic.

**Methods:**

A web survey was conducted between April and May 2020 in both countries. The main outcome was a positive screening for depression only, anxiety only, or both. Lifestyle was measured using a lifestyle multidimensional scale adapted for the COVID-19 pandemic (Short Multidimensional Inventory Lifestyle Evaluation–Confinement). A multinomial logistic regression model was performed to evaluate the factors associated with depression, anxiety, and the presence of both conditions.

**Results:**

From the 22,786 individuals included in the web survey, 3745 self-reported to be essential workers. Overall, 8.3% (n=311), 11.6% (n=434), and 27.4% (n=1027) presented positive screenings for depression, anxiety, and both, respectively. After adjusting for confounding factors, the multinomial model showed that an unhealthy lifestyle increased the likelihood of depression (adjusted odds ratio [AOR] 4.00, 95% CI 2.72-5.87), anxiety (AOR 2.39, 95% CI 1.80-3.20), and both anxiety and depression (AOR 8.30, 95% CI 5.90-11.7). Living in Brazil was associated with increased odds of depression (AOR 2.89, 95% CI 2.07-4.06), anxiety (AOR 2.81, 95%CI 2.11-3.74), and both conditions (AOR 5.99, 95% CI 4.53-7.91).

**Conclusions:**

Interventions addressing lifestyle may be useful in dealing with symptoms of common mental disorders during the strain imposed among essential workers by the COVID-19 pandemic. Essential workers who live in middle-income countries with higher rates of inequality may face additional challenges. Ensuring equitable treatment and support may be an important challenge ahead, considering the possible syndemic effect of the social determinants of health.

## Introduction

The prevalence of COVID-19 continues to increase in Brazil [1], and mental health is recognized as an important challenge ahead worldwide [2-4]. Several studies have used web surveys to screen for common mental health disorders (mainly depressive and anxiety disorders) among the general population [5-8]. Some of these studies, but not all [9,10], have shown that essential workers, such as health care workers (HCWs), had an increased likelihood of anxiety and depression compared to other workers [11,12]. Essential workers may have increased workload and working hours during the pandemic [13], struggle with the lack of adequate personal protective equipment [14], and may be isolated from friends and families [15]. Burnout symptoms [16], emotional exhaustion [17], and fear of transmitting the virus [18] are commonly reported.

The first study investigating mental health problems among essential workers during the COVID-19 pandemic was conducted in China and found the prevalence of anxiety and depressive symptoms to be at 20.1% and 12.7%, respectively [19]. In two subsequent systematic reviews, HCWs presented increased depression/depressive symptoms, anxiety, psychological distress, and poor sleep quality [20,21]. The first meta-analysis evaluating the prevalence of mental health problems among HCWs (up to April 13, 2020) found 13 studies (N=33,062 participants), of which 12 were conducted in China. The pooled prevalence of a positive screening for anxiety was 23.2% (95% CI 17.8-29.1) while depression was estimated at 22.8% (95% CI 15.1-31.5) [22]. Evidence from outside of China is still scarce [23-30], and it is difficult to estimate the overall prevalence of common mental health disorders due to methodological issues, heterogeneity in study populations and sizes, and differences in criteria used to define a case (eg, different instruments and cut-offs). For instance, the prevalence of depression was as low as 10% among HCWs in Singapore and India [23] to as high as 64.7% among physicians in Turkey [31].

Four major groups of variables have been associated with an increased likelihood of having a positive screening for anxiety and/or depression among essential workers during the COVID-19 pandemic: demographic, professional/financial worries, COVID-19 exposure factors, and personal health factors. In terms of demographics, being female and younger were more frequently associated with depression [31,32] while a higher education level and residing in areas or provinces with a greater number of cases have been associated with anxiety [19,33,34]. Worrying about adequate training, knowledge, preparedness, and finances, as well as self-efficacy and career phase, were some of the professional/financial variables analyzed under this domain [34]. COVID-19 exposure variables included being a frontline worker [31,33,34]; fear, suspicion, or diagnosis of COVID-19 for oneself and/or their significant other [19,32,33,35]; and having a deceased colleague [32]. Finally, regarding personal health factors, included variables were perceived stress [19], poor sleep [19,26], presence of a previous medical or psychiatric disorder [31,35], and a history of alcohol consumption [35]. Of note, good social support was frequently associated with a decreased likelihood of anxiety and depression among essential workers [19,36].

The fast growing field of lifestyle psychiatry [37] has been providing evidence on how health behaviors (eg, diet, physical activity, smoking, and sleep) relate to the prevalence, incidence, and adverse outcomes of mental health disorders [38-40]. For instance, consistent results point to the beneficial effects of physical activity in preventing the onset of depression as well as improving its symptoms [40]. As many people around the world are under confinement due to the COVID-19 pandemic, changes in lifestyle behaviors have attracted more research interest [41]. Behaviors, such as sleep quality [36,42], were assessed among HCWs instead of using a comprehensive, multidimensional approach to lifestyle. Multidimensional evaluations of lifestyle are still scarce [41], although it is possible that different health behaviors share a common pathway to improve mental health, such as anti-inflammatory effects [43,44]).

**Figure 1:**
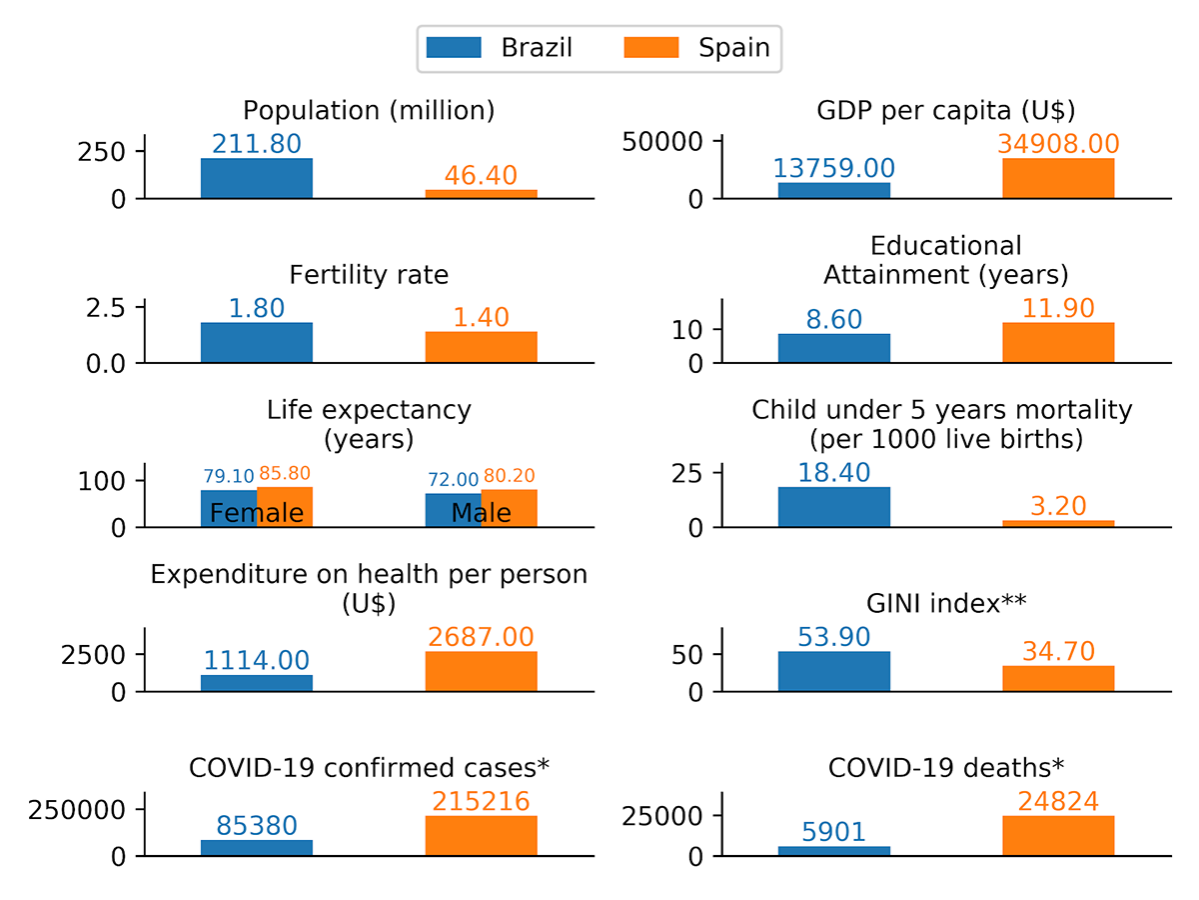
Select social and health indicators (2017) and the COVID-19 situation (as of May 2, 2020) for Brazil and Spain. GDP: gross domestic product. Data sources: Institute from Health Metrics and Evaluation [47], *World Health Organization [48,49], **World Bank 2017-2018 [50].

Considering the social determinants of health [45,46], it is possible that countries presenting poor social and health indicators may present a higher prevalence of unhealthy outcomes, which could include both COVID-19–related and mental health problems. Herein, we have included two countries with different social and health indicators, and at different stages of the COVID-19 pandemic, as summarized in Figure 1 [47-50]. Brazil has roughly 4.5 times the population of Spain, but 0.4 times the GDP (gross domestic product) and 0.4 times the expenditure on health. In addition, Brazil is considered one of the countries with the greatest inequalities in income/wealth globally, with a Gini index of 53.9. The first COVID-19 case was diagnosed on January 31, 2020, in Spain and on February 26, 2020, in Brazil, respectively. On May 2, 2020 (the midpoint of our data collection period), there were almost 25,000 deaths in Spain and 6000 deaths in Brazil. At that time, Spain was under a strict lockdown policy while the lockdown in Brazil was implemented partially and in select cities and counties.

So far, we are unaware of studies that have investigated mental health problems among essential workers from two countries presenting such different social and epidemic profiles. Thus, our major aim is to describe the prevalence of depression, anxiety, and the comorbidity of both, as well as their associated factors, among self-reported essential workers during the COVID-19 pandemic in Brazil and Spain.

## Methods

### Study Design

A web survey was conducted from April 15 to May 15, 2020, in Spain and April 20 to May 20, 2020, in Brazil. The online questionnaire was created using SurveyGizmo and included questions about demographics, COVID-19 experience, lifestyle behaviors, self-rated health, and previous diagnosed conditions. The questionnaire comprised 101 questions, and skips, when appropriate, were implemented to decrease the time of completion (Multimedia Appendix 1). The usability and technical functionality were tested before launching the survey in both countries. In addition, participants could read information regarding ways to maintain a healthy lifestyle during the pandemic while they were answering the questionnaire, and were provided with additional websites and telephone numbers to find reliable information regarding COVID-19. This information was compiled from the COVID-19–related webpages of the Centers for Disease Control and Prevention, the National Institute of Health (United States), the Oswaldo Cruz Foundation, the Brazilian Ministry of Health, and the Spanish Ministry of Health.

### Study Population

**Figure 2:**
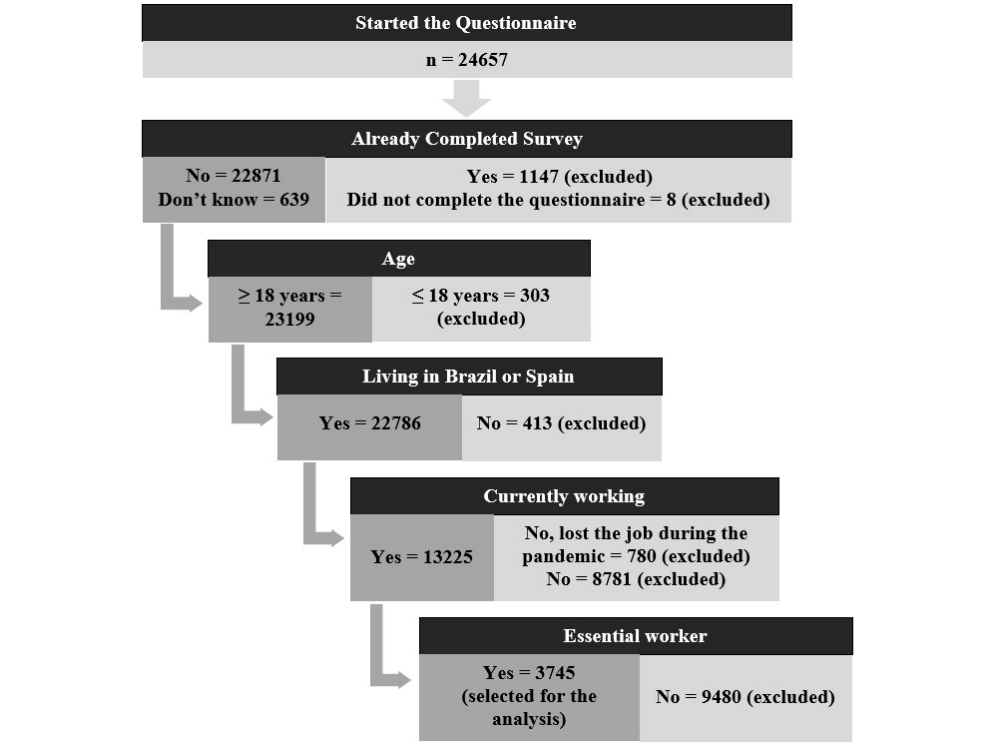
Inclusion flowchart.

The study population included adults from both sexes living in Spain or in Brazil, who had access to the internet, and agreed to participate in the study after reading the informed consent form. Herein, we selected individuals who reported to be essential workers. Essential workers were considered all participants who answered “yes” to the following question: “Are you currently working as a health care worker or as a professional of other essential services (transportation, food, cleaning)?” To avoid duplicated responses, individuals who reported having previously completed the questionnaire were excluded (Figure 2) as no identification data (nor IP [Internet Protocol] address) were collected.

### Sample and Recruitment

**Figure 3:**
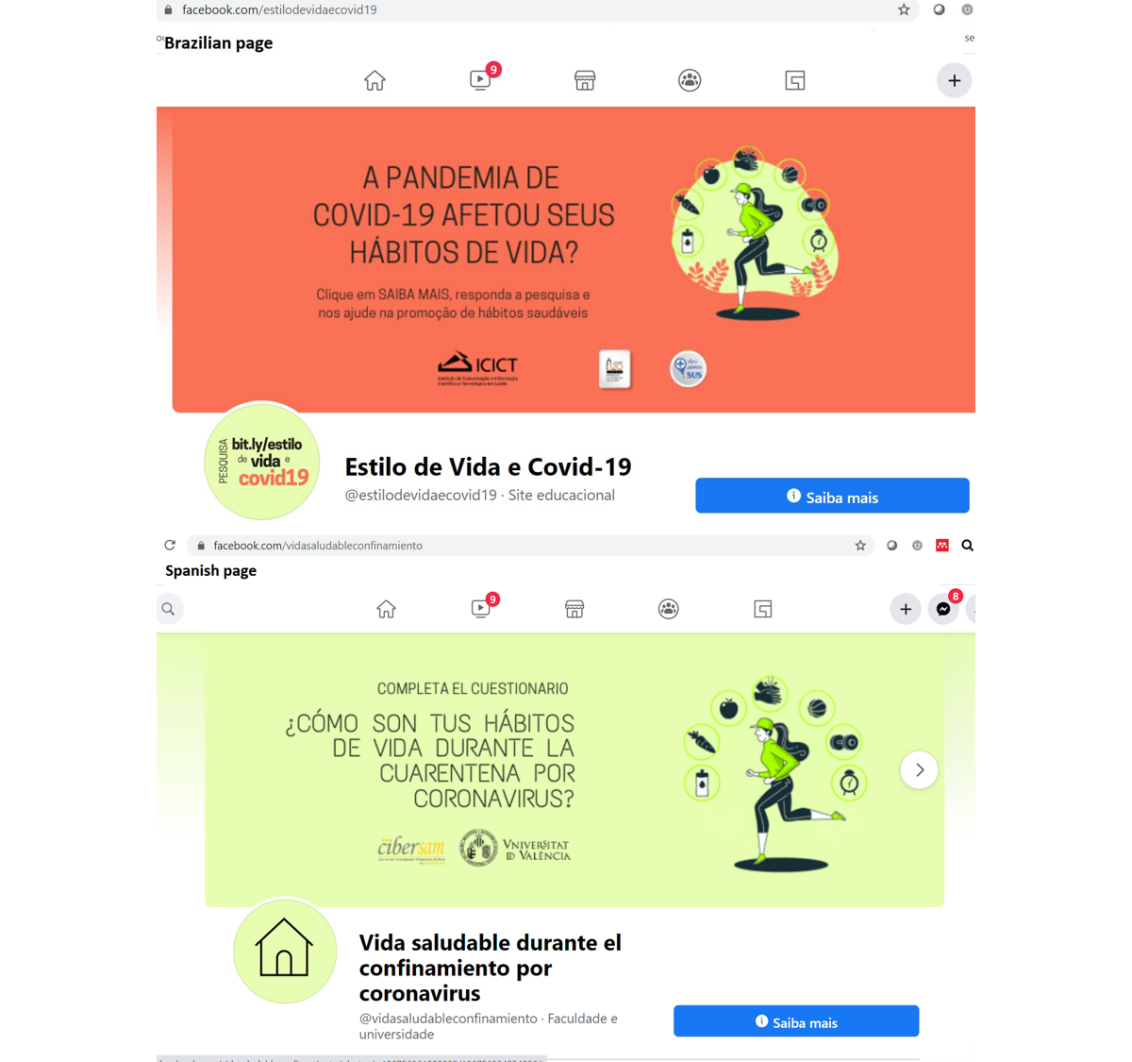
Facebook pages created to disseminate the project and healthy lifestyle behaviors during the COVID-19 pandemic in Brazil and Spain.

A convenience sample of participants was recruited via social networks (Facebook, WhatsApp, and Twitter) using a snowball technique and sponsored social network advertisements. A Facebook page for each country (Figure 3) was created and boosted using the following words: “healthy lifestyles,” “sad,” “happiness,” “fear,” “emotion,” “stress,” “well-being,” “self-esteem,” “quality of life,” “motivation,” “mind,” “boredom,” “panic,” “interpersonal relationship,” “life,” “emotional intelligence,” “physical fitness,” and “physical exercise.” According to Facebook, the page would reach 87 million people in Brazil and 290,000 individuals in Spain. Fundamental parameters were unknown when the sample size was defined, such as the possible participation of superrecruiters [51] with the potential to skew the sampling process, or whether or not there would be structural bottlenecks. Therefore, the sample size was not defined a priori*;* instead, a 30-day period of data collection was specified.

### Response Rates

Response rates were not estimated in the study since the study denominator is unknown (ie, we were unable to estimate how many individuals had send the survey link via different—and probably overlapping—social networks).

### Variables and Measurements

#### Study Outcome (Dependent Variable)

The main outcome is a positive screening for depression and/or anxiety. Depression was screened using the Patient Health Questionnaire-2 (PHQ-2 [52-55]; cut-off ≥3), and anxiety was screened using the Generalized Anxiety Disorder 7-item scale (GAD-7 [56]; cut-off ≥10). Subsequently, two dichotomous variables were created: “Positive Depression” and “Positive Anxiety.” The outcome was a composite variable created using the aforementioned variables with the following categories: negative screening for both depression and anxiety, positive screening for depression only, positive screening for anxiety only, and positive screening for both depression and anxiety.

#### Independent Variables

Demographic information included sex, age, educational level (aggregated as primary/secondary education, a professional degree, a university degree, or a master’s/PhD degree), number of people living in the household, frontline worker (yes/no), and country of residence (Brazil/Spain). Social distancing/self-isolation was considered as a dichotomous variable (yes/no).

Questions related to COVID-19 were as follows: “Have you been diagnosed with COVID-19?”; “Have you been admitted to a hospital or hospitalized due to COVID-19?”; and “Have you lost a significant other?” Possible answers were yes or no.

Lifestyle habits were assessed using the Short Multidimensional Inventory Lifestyle Evaluation–Confinement (SMILE-C) [41]. This scale was developed specifically to allow a multidimensional measure of lifestyle during the COVID-19 pandemic. It comprises 27 items made up of 7 domains (diet and nutrition, substance abuse, physical activity, stress management, restorative sleep, social support, and environmental exposures), with response options measured using a 4-point Likert scale. The SMILE-C has an overall Cronbach α=0.75 and Kaiser-Meyer-Olkin Measure=0.77. The higher the score, the healthier the lifestyle pattern. In this study, the SMILE-C total score was dichotomized at the 75th percentile (up to 85%).

Self-rated health was measured using the question “How would you rate your health in general?” with possible answer choices of “very bad,” “bad,” “neither good nor bad,” “good,” and “very good” [57]. Response options were aggregated into very good/good and neither good nor bad/bad/very bad.

Previously diagnosed conditions were investigated using the question “In the last 12 months, have you been diagnosed by a medical doctor or health professional, or received treatment for any of the following conditions?” Conditions included diabetes, heart disease, hypertension, stroke, anemia, asthma, depression, anxiety, bipolar disorder, schizophrenia, anorexia/bulimia, HIV/AIDS, cancer, tuberculosis, cirrhosis, and renal disease [58]. The conditions were then aggregated as chronic diseases, mental health disorders, and infectious diseases.

Screening for alcohol abuse was performed using the Alcohol Use Disorder Identification Test (AUDIT-C; cut-off ≥3 [59]).

Changes in the SMILE-C domains during the pandemic were evaluated using questions like “Did you change your nutritional habits and diet during the COVID-19 pandemic?” Response options were measured using a 4-point Likert scale (completely, moderately, mildly, not at all) and aggregated into completely/moderately and mildly/not at all.

### Statistical Analysis

Nonresponse treatment is described in Multimedia Appendix 2. Independent variables were described by outcome and proportions compared using chi-squared tests. Taking into consideration the complex, multiple associations of the different covariates with the outcomes under analysis (screening for depression and anxiety), preliminary analyses using Least Absolute Shrinkage and Selection Operator (LASSO [60, 61]), a simple machine learning procedure, were employed. LASSO is a penalized regression analysis method that helps to optimize variable selection and regularization in order to enhance the accuracy of the model to be implemented. The subset of factors (variables) that did not contribute to the hypothetical model under assessment yield zero coefficients [62]. Such variables were excluded from subsequent multivariable analyses (Multimedia Appendix 2).

The second procedure to optimize variable selection was based on the simulation of different modeling strategies with the subsequent choice of the best subset of variables to be included in a parsimonious model based on the best R^2^ coefficient [63, 64]. The glmnet library and the regsubsets function from R 4.0.2 (The R Foundation) were used.

Using this subset of variables, a multinomial logistic regression was fitted, taking as the reference category “negative screening for both depression and anxiety.” Analyses used the backward strategy, with the progressive elimination of variables based on the results of the Wald test and maximum likelihood estimation of fitness, considering a significance level of 5.0%. The model fitness was evaluated using different diagnostic tools, such as the Hosmer-Lemeshow statistics, the Pearson chi-squared test, as well as the deviance information criterion. Additionally, an analysis of residuals was performed (Multimedia Appendix 2). The model yielded adjusted odds ratios (AORs) with their respective 95% CIs.

### Ethical Aspects

The study was approved by the Ethics Committee at the Hospital Universitari i Politècnic La Fe in Valencia, Spain, and by the Comissão Nacional de Ética em Pesquisa (CONEP, Brazil – 3.968.686). The survey was anonymous (no identifying data like name, zip code, or IP address were collected), and participants read the consent form and confirmed their interest in participating before starting the questionnaire. As mentioned before, as a direct benefit, participants were provided with tips on healthy lifestyles and reliable websites and telephone numbers for additional information regarding COVID-19.

## Results

Overall, 24,657 questionnaires were initiated, and 22,786 were eligible for the main study. Of those, 3745 reported to be working as an essential worker during the COVID-19 pandemic and comprised the study sample (Figure 2). In total, 2842 participants were from Brazil and 903 from Spain. Most were female (Brazil: n=2052, 72.2%; Spain: n=640, 70.9%; *P*=.44), with a median age of 39 (IQR 32-51) years for Brazil versus 43 (IQR 32-52) years for Spain (*P*=.07). Half of the sample (n=457, 50.6%) reported being a frontline personnel in Spain compared to 28.9% (n=822) in Brazil (*P*<.001) (Table 1).

**Table 1:** Demographics, COVID-19 experience, lifestyle, and self-reported health conditions by country among essential workers from Brazil and Spain (N=3745), April to May 2020.

Variable		Brazil (n=2842), n (%)	Spain (n=903), n (%)	Total (N=3745), n (%)	*P* value
**Screening for depression and/or anxiety**					<.001
	Negative for both depression and anxiety	1280 (45.0)	693 (76.7)	1973 (52.7)	
	Positive for depression only	262 (9.2)	49 (5.4)	311 (8.3)	
	Positive for anxiety only	360 (12.7)	74 (8.2)	434 (11.6)	
	Positive for both depression and anxiety	940 (33.1)	87 (9.6)	1027 (27.4)	
**Sex**					.44
	Male	790 (27.8)	263 (29.1)	1053 (28.1)	
	Female	2052 (72.2)	640 (70.9)	2692 (71.9)	
Age (years), median (IQR)		39 (32-51)	43 (32-52)	40 (32-51)	.07
**Educational level**					<.001
	Primary/secondary education or professional degree	425 (15.0)	242 (26.8)	667 (17.8)	
	University degree	1606 (56.5)	354 (39.2)	1960 (52.3)	
	Master’s/PhD degree	811 (28.5)	307 (34.0)	1118 (29.9)	
Frontline worker		822 (28.9)	457 (50.6)	1279 (34.2)	<.001
People living in the household, median (IQR)^a^		3 (2-4)	3 (2-4)	3 (2-4)	.56
Self-isolated^b^		1501 (53.3)	181 (20.2)	1682 (45.3)	<.001
Diagnosed with COVID-19^c^		69 (2.4)	35 (3.9)	104 (2.8)	.02
Lost someone during the pandemic^c^		254 (9.0)	97 (10.8)	351 (9.4)	.10
SMILE-C^d^, median (IQR)		78 (71-84)	80 (75-85)	79 (72-85)	<.001
Self-reported health (neither good nor bad, bad or very bad)^e^		708 (24.9)	210 (23.3)	918 (24.5)	.32
**Diagnosed with or treated for…**					
	Chronic diseases^f^	929 (32.9)	233 (26.1)	1162 (31.3)	<.001
	Mental health disorders^g^	865 (30.9)	109 (12.2)	974 (26.4)	<.001
	Infectious diseases^h^	108 (3.8)	3 (0.3)	111 (3.0)	<.001
Positive screening for alcohol abuse		1260 (44.3)	289 (32.0)	1549 (41.4)	<.001
**Changes in…**						
	Dietary and nutritional habits^i^	1257 (44.3)	217 (24.0)	1474 (39.4)	<.001
	Substance use habits^j^	459 (17.6)	83 (9.3)	542 (15.5)	<.001
	Physical activity routine^k^	1656 (58.8)	584 (64.7)	2240 (60.2)	.002
	Strategies to manage stress^l^	1530 (53.9)	281 (31.2)	1811 (48.5)	<.001
	Sleep patterns^i^	1219 (42.9)	243 (26.9)	1462 (39.1)	<.001
	Social support^m^	1543 (54.8)	336 (37.6)	1879 (50.6)	<.001
	Time spent indoors/outdoors^c^	2461 (86.7)	823 (91.5)	3284 (87.9)	<.001

^a^n=2

^b^n=30

^c^n=7

^d^SMILE-C: Short Multidimensional Inventory Lifestyle Evaluation-Confinement; the higher the score, the healthier the lifestyle.

^e^n=5

^f^n=31

^g^n=56

^h^n=6

^i^n=3

^j^n=242

^k^n=25

^l^n=8

^m^n=33

The prevalence of positive screenings for depression, anxiety, and comorbidity of both was 8.3% (n=311), 11.6% (n=434), and 27.4% (n=1027), respectively. All were higher in Brazil compared to Spain (Table 1). Table 2 describes the sociodemographic and clinical characteristics of the sample across the outcome categories (negative for depression and anxiety, positive for depression only, positive for anxiety only, and positive for both depression and anxiety).

**Table 2:** Demographics, COVID-19 experience, lifestyle, and self-reported health conditions by mental health outcomes among essential workers from Brazil and Spain (N=3745), April to May 2020.

Variable		Negative for both depression and anxiety (n=1973)	Positive for depression only (n=311)	Positive for anxiety only (n=434)	Positive for depression and anxiety (n=1027)	*P* value
**Country** **, n (%)**						<.001
	Brazil	1280 (64.9)	262 (84.2)	360 (82.9)	940 (91.5)	
	Spain	693 (35.1)	49 (15.8)	74 (17.1)	87 (8.5)	
**Sex** **, n (%)**						<.001
	Male	619 (31.4)	91 (29.3)	112 (25.8)	231 (22.5)	
	Female	1354 (68.6)	220 (70.7)	322 (74.2)	796 (77.5)	
Age (years) , mean (SD)		44.56 (12.33)	39.25 (11.78)	41.10 (10.63)	37.43 (10.96)	<.001
**Educational level** **, n (%)**						<.001
	Primary/secondary education or professional degree	340 (17.2)	43 (13.8)	62 (14.3)	222 (21.6)	
	University degree	984 (49.9)	177 (56.9)	228 (52.5)	571 (55.6)	
	Master’s/PhD degree	649 (32.9)	91 (29.3)	144 (33.2)	234 (22.8)	
Frontline worker, n (%)		660 (33.5)	83 (26.7)	172 (39.6)	364 (35.4)	.002
People living in the household, median (IQR)^a^		3 (2-4)	3 (2-4)	3 (2-4)	3 (2-4)	.008
Self-isolated^b^, n (%)		824 (41.9)	163 (53.1)	191 (44.5)	504 (49.7)	<.001
Diagnosed with COVID-19^c^, n (%)		45 (2.3)	8 (2.6)	18 (4.2)	33 (3.2)	.13
Lost someone in the pandemic^c^, n (%)		169 (8.6)	24 (7.7)	48 (11.1)	110 (10.7)	.11
SMILE-C^d^, mean (SD)		82.23 (7.71)	76.16 (7.73)	77.67 (7.43)	71.41 (8.73)	<.001
Self-reported health (neither good nor bad, bad, or very bad)^e^		280 (14.2)	77 (24.8)	112 (25.8)	449 (43.8)	<.001
**Diagnosed with or treated for…**						
	Chronic diseases^f^	568 (29.1)	75 (24.2)	140 (32.4)	379 (37.3)	<.001
	Mental health disorders^g^	270 (13.8)	74 (24.0)	129 (30.3)	501 (50.4)	<.001
	Infectious diseases^h^	50 (2.5)	8 (2.6)	14 (3.2)	39 (3.8)	.26
Positive screening for alcohol abuse		736 (37.3)	143 (46.0)	195 (44.9)	475 (46.3)	<.001
**Changes in…**						
	Dietary and nutritional habits^i^	641 (32.5)	145 (46.6)	207 (47.7)	481 (46.9)	<.001
	Substance use habits^j^	213 (11.4)	50 (17.4)	64 (16.2)	215 (22.5)	<.001
	Physical activity routine^k^	1187 (60.4)	193 (62.9)	287 (66.4)	573 (56.4)	.003
	Strategies to manage stress^l^	905 (45.9)	157 (50.6)	255 (58.9)	494 (48.2)	<.001
	Sleep patterns^i^	540 (27.4)	133 (42.8)	203 (46.9)	586 (57.1)	<.001
	Social support^m^	892 (45.6)	158 (51.6)	240 (55.6)	589 (58.0)	<.001
	Time spent indoors/outdoors^c^	1756 (89.1)	269 (86.5)	381 (88.0)	878 (85.7)	.049

^a^ n=2

^b^n=30

^c^ n=7

^d^SMILE-C: Short Multidimensional Inventory Lifestyle Evaluation-Confinement; the higher the score, the healthier the lifestyle.

^e^n=5

^f^n=31

^g^n=56

^h^n=6

^i^n=3

^j^n=242

^k^n=25

^l^n=8

^m^n=33

**Figure 4:**
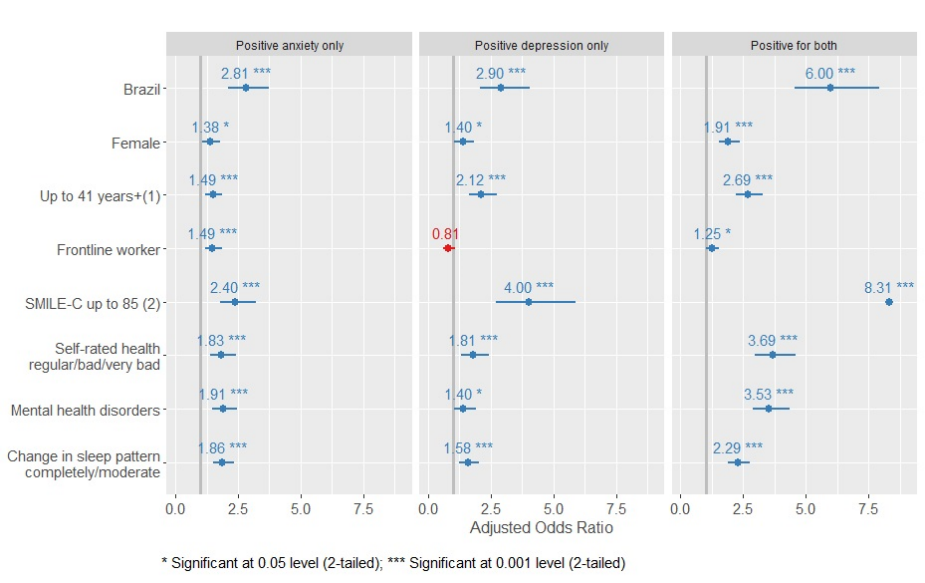
Factors associated with depression or anxiety or both (multinomial model) among essential workers from Brazil and Spain (n=3745). April-May, 2020. Note: (1) Age was dichotomized by the sample median age. (2) The SMILE-C (Short Multidimensional Inventory Lifestyle Evaluation-Confinement was dichotomized at the percentile 75%, the higher the score, the healthier the lifestyle).

In the multinomial model, living in Brazil was associated with an AOR of 2.89 (95% CI 2.07-4.06) for a positive screening for depression, an AOR of 2.81 (95% CI 2.11-3.74) for anxiety, and an AOR of 5.99 (95% CI 4.53-7.91) for both conditions compared to living in Spain. An unhealthy lifestyle was associated with an AOR of 4.00 (95% CI 2.72-5.87) for depression, an AOR of 2.39 (95% CI 1.80-3.20) for anxiety, and an AOR of 8.30 (95% CI 5.90-11.7) for both conditions. Interestingly, being a frontline worker was not associated with depression only (AOR 0.81, 95% CI 0.60-1.08), although it increased the likelihood of having anxiety (AOR 1.49, 95% CI 1.18-1.87) and both conditions (AOR 1.25, 95% CI 1.02-1.53). Additionally, we found that being female, being younger, presenting moderate or substantial changes in sleep patterns during the COVID-19 pandemic, being diagnosed with or treated for mental health disorders in the last year, and reporting a reduction in self-rated health were all associated with a higher likelihood of having depression, anxiety, or both conditions (Figure 4 and Multimedia Appendix 2).

## Discussion

In a web survey of 3745 essential workers from Brazil and Spain, we showed 8.3%, 11.6%, and 27.4% presenting positive screenings for depression, anxiety, and both conditions, respectively. As in previous studies conducted during the COVID-19 pandemic, we found that women, younger workers, frontline workers, those reporting a mental health diagnosis or treatment in the last 12 months, and those reporting changes in sleep patterns presented a higher likelihood of a positive screening for anxiety and/or depression. Most importantly, higher odds ratios were observed in those living in Brazil and in those following an unhealthy lifestyle.

During data collection, Spain had 4 times the number of COVID-19 deaths than Brazil and had adopted a strict lockdown policy. It could be expected that essential workers under these conditions would be more prone to present anxiety and depressive symptoms. However, Brazil has additional social, structural, and political problems that may affect mental health. Recently, Baqui et al [65] reported on the higher mortality risk among “pardo” and Black Brazilians admitted to hospital due to COVID-19; ethnicity was the second most important risk factor for death (after age). In addition, the authors highlighted that comorbid diseases and death were more common among Brazilians from the North region compared to the Central-South (except for Rio de Janeiro). Ribeiro et al [66] highlighted that the worst public health and socioeconomic scenarios were present in the northern regions of Brazil; higher proportions of individuals living in substandard housing (slums), with reduced schooling and a lack of sanitation and piped water, may interfere with adherence to hygiene recommendations. Although both papers discussed intracountry inequalities, social and health inequalities may be even higher between different countries [67] (Figure 1). In addition, the political instability and the government’s failure to acknowledge the seriousness of the pandemic (eg, official data on COVID-19 was not being published) [68] may be worsening the consequences of COVID-19 in Brazil, including repercussions concerning the mental health of essential workers. These workers may be seeing a high number of casualties, and working under intense fear and feelings of impotence, which may be related to the higher odds for anxiety and depression observed in our study.

Self-reported unhealthy lifestyle behaviors during confinement were associated with an increased likelihood of presenting a positive screening for both anxiety and depression in our study. Several studies have assessed psychological distress in HCWs during COVID-19, but its association with lifestyle remains underresearched. To our knowledge, the present study is the first web survey designed to assess a wide range of lifestyle changes and its relationship with anxiety and depression among essential workers during the pandemic. Among HCWs from New York, where almost half screened positive for depression, and one third for anxiety [30], physical activity/exercise was the most commonly endorsed solution to cope with COVID-19–related psychological distress, but its relationship with anxiety and depression was not explored. Our results are consistent with those pertaining to the general population and clinical studies. For instance, in Australia, adults who reported negative changes in physical activity, sleep, smoking, and alcohol intake after the onset of COVID-19 were more likely to have higher rates of depression, anxiety, and stress symptoms [69]. In the same way, individuals with anxiety and depression have shown higher ratios of unhealthy lifestyle habits, including poor diet quality, impaired sleep, reduced physical activity, smoking, and substance and/or alcohol misuse [39]. Based on the present results, the relationship between anxiety/depression and lifestyle as a multidimensional construct applies also to essential workers during the COVID-19 pandemic.

Our findings regarding sleep changes are also in accordance with a meta-analysis that showed that about 50% of HCWs have poor sleep quality in general (ie, during nonpandemic times) [36]. Subjective sleep quality, defined by the satisfaction with one’s overall sleep experience, may worsen among frontline HCWs treating patients with COVID-19 [42]. The present study expands the association between changes in sleep and anxiety/depression to a wider group of essential workers. This concurs with evidence supporting a bidirectional relationship between sleep disturbances and anxiety/depression [70]. Moreover, a reduced quality of sleep was associated with higher levels of depressive and anxiety symptoms during the COVID-19 lockdown in Italy [71].

Our results are in accordance with most of the literature: women and youth [17,31,32,72], frontline workers [31,33,34], a diagnosis of or treatment for mental health disorders in the past 12 months, and self-rated poor health [17] all increased the likelihood of depression and anxiety. Mental health disorders are associated with higher mortality rates and shorter life expectancies [73]. Our results, which support that of The Lancet Commission on Global Mental Health and Sustainable Development [74], showed the importance of social and environmental factors in mental health and highlighted the additional challenges experienced by populations living in countries with higher rates of inequality.

Consistent with other web surveys, where the population is recruited through social networks, our sample is not probabilistic and may therefore not represent the entire population of essential workers from Brazil and Spain [75]. Additionally, in 2018, 67% of Brazilian households had internet access (48% among the lower economic strata) [76] compared to 86.4% in Spain [77]. Although this difference may not have contributed to the different prevalence found between the countries (as we may have surveyed individuals with age, income, and schooling more similar to the Spanish population), we may be overrepresenting the highest socioeconomic strata in Brazil. Women were also overrepresented in both countries, as in many other web surveys conducted during the COVID-19 pandemic [18,23,32,34,72]. Due to the length of the original questionnaire, we did not include questions regarding gender and ethnicity/race, which may be associated with higher vulnerability to COVID-19 [65] and mental health outcomes. Additionally, we did not ask about the specific profession of essential workers and were not able to assess which professional groups were more vulnerable to mental health problems. In the United States [78], over 75% of Americans were estimated to work in occupations (including health care, manufacturing, retail, and food services) that are challenging to do from home. It was suggested that those workers may receive low wages and be subjected to stress due to the lower income and job insecurity, which could result in a large burden of mental health disorders.

One important strength of our survey was to disseminate reliable information on COVID-19 and strategies to maintain a healthy lifestyle. Considering the massive amount of information available, including fake news, and all the technology available for creating and disseminating online surveys, we believe that researchers can contribute to society by providing valuable information to respondents while obtaining data. Studies addressing participants’ opinions and effectiveness, when appropriate, on this information should be considered in the future.

Finally, our results provide additional support for The Lancet COVID-19 Commission [79], and are in accordance with Vigo et al [80], showing that countries with higher rates of inequality may be facing an important mental health burden in the forthcoming months.

## Appendix

Multimedia Appendix 1 Data collection.

Multimedia Appendix 2 Statistical analyses.

## Abbreviations

AOR: adjusted odds ratio
AUDIT-C: Alcohol Use Disorder Identification Test
CNPq: Conselho Nacional de Desenvolvimento Científico e Tecnológico
CONEP: Comissão Nacional de Ética em Pesquisa
FAPERJ: Fundação de Amparo à Pesquisa do Estado do Rio de Janeiro
GAD-7: Generalized Anxiety Disorder 7-item
GDP: gross domestic product
HCW: health care worker
IP: Internet Protocol
LASSO: Least Absolute Shrinkage and Selection Operator
PHQ-2: Patient Health Questionnaire-2
SMILE-C: Short Multidimensional Inventory Lifestyle Evaluation–Confinement
