# Supplementary material for: Depression, Anxiety, and Lifestyle Among Essential Workers: A Web Survey From Brazil and Spain During the COVID-19 Pandemic
Source: J Med Internet Res. 2020 Oct 30;22(10):e22835. doi: 10.2196/22835 (PMC7641648; doi:10.2196/22835)
Supplement: Multimedia Appendix 1 [file jmir_v22i10e22835_app1.pdf]

DATA COLLECTION FORM

Informed consent

The Instituto de Comunicação e Informação Cientifica e Tecnológica em Saúde / FIOCRUZ is performing the online survey "**HEALTHY HABITS AND LIFESTYLE DURING THE COVID-19 PANDEMIC: A WEBSURVEY FOR THE BRAZILIAN POPULATION**" to assess how the COVID-19 pandemic is affecting the lifestyle and well-being of individuals in Brazil. This is important to evaluate the need for dissemination of new information and plan health services in our country.

For this reason, we are inviting men and women aged 18 or older, living in any city of Brazil to answer this online questionnaire, which lasts 10 to 15 minutes.

As a benefit, when filling out the questionnaire, you will see recommendations on how to keep a healthy lifestyle during and after the pandemic. In addition, as an indirect benefit, you will be contributing to the planning of interventions for the Brazilian population.

Important information:

1. Your answers are **anonymous and cannot be tracked**: we will have no information about who you are apart from the questions you answer.
2. Some questions are related to **sensitive topics**. You **may refuse to answer** any questions you are not comfortable about answering.
3. **Your participation is voluntary** and you may withdraw the study at any moment, and it will not cause any problems/consequences to you.
4. If you have any questions about the research now or later, please contact the researchers responsible for this survey Dra. Raquel B. De Boni (raqueldeboni@gmail.com) and Dr. Francisco Inácio Bastos (francisco.inacio.bastos@hotmail.com). You can also contact the ethics committee that approved this project (Comitê de Ética em Pesquisa com Seres Humanos da Escola Politécnica Joaquim Venâncio - cep.epsjv@fiocruz.br).

I state that I have read and understood the Informed Consent Form and I accept to participate in the research. (The participant must check the box ▢ to access the questionnaire).

The COVID-19 is a new disease and new discoveries are being made every time. We all should keep hygiene habits and follow the recommendations of the World Health Organization, and local authorities. This is the best way to help reduce transmission.

If you have questions about COVID-19, search and share only reliable and up-to-date information on websites of institutions such as the World Health Organization (https://www.who.int/emergencies/diseases/novel-coronavirus-2019), the ministry of health (https://coronavirus.saude.gov.br), and Fiocruz (https://portal.fiocruz.br/coronavirus)

Q1. Have you participated in this online survey at any point?

0. No

1. Yes → "Thank you for your interest. We are only interviewing each participant one time." [the questionnaire ends.]

2. I don't know

Q2. What is your sex at birth?

1. Male
2. Female

Q3. How old are you?

_____ Years-old (If age < 18 years-old: " Thank you for your interest .We are only interviewing individuals 18 years-old or older at this time." [the questionnaire ends.]

Q4. Do you live in Brazil?

1. Yes

0. No → "Thank you for your interest, we are only interviewing individuals living in Brazil at this time." [the questionnaire ends.]

Q5. In which state are you living? [open box with the states]

Q6. Do you live in the capital city of your state?

1. Yes

2. No. Which city do you live in? ________________________

Q7. Are you currently working?

1. Yes

0. No →SKIP TO Q10

2. I lost my job during the pandemic → SKIP TO Q11

Q8. Are you currently working as a healthcare worker or as a professional of other essential services (transportation, food, cleaning)?

1. Yes

0. No → SKIP TO Q11

Q9. Are you currently working in the front line?

1. Yes

0. No → SKIP TO Q11

Q10. Are you currently studying?

1. Yes

0. No

Q11. How many years of education* have you completed? ________ years of education

*The elementary school lasts 8 or 9 years, the high school and technical education usually lasts 3 years. A university degree lasts 4 – 6 years, the master's degree lasts 2 years, and the doctorate degree lasts 4 years.

Q12.What's the highest degree of education you have achieved?

1 Elementary
3. High School
4. Technical education
5. University
6. Masters/PhD

Q13. How many people live in your household right now, including yourself? ___________ people

Q14. Are you on self-isolation at this moment?

1.Yes → SKIP TO Q16
0.No
999. I prefer do not answer

Q15. Were you on self-isolation at some point during the COVID-19 pandemic?

1.Yes
0.No
999. I prefer do not answer

Q16. How long have you been self-isolated? ________ number of days

Q17. Did a health professional formally diagnose you with COVID-19?

1.Yes
2.No → SKIP TO Q20
999. I prefer do not answer

Q18. Were you admitted to a hospital?

1.Yes
2.No
999. I prefer do not answer

Q19. Were you on mechanical ventilation?

1.Yes
2.No
999. I prefer do not answer

Q20. Have you lost a significant one during the pandemic?

1.Yes
2.No
999. I prefer do not answer

In the last month, how often in your daily routine…	(1) Always	(2) Often	(3) Seldom	(4) Never	(999) I prefer not to answer
**Q21. Do you eat meals you or someone else in your family prepares?**					

**Q22. When shopping for food, do you check labels for ingredients such as quantity of salt?**					
**Q23. Do you eat processed food (frozen food such as pizza, French fries, puff pastries, deep-fried foods and canned foods)?**					
**Q24. Do you eat fast-food, high-calorie sweet or fatty foods when you are stressed or sad?**					
**Q25. Do you eat healthy foods such as fresh fruits, fresh vegetables, wholegrain, legumes or nuts?**					
**Q26. Do you keep a regular meal schedule?**					
**Q27. Do you share your main meals with friends or family?**					

Q28. Have you changed your dietary and nutritional habits during the COVID-19 pandemic?
(1) Completely (2) Moderately (3) Slightly (4) Not at all (999) I prefer do not answer

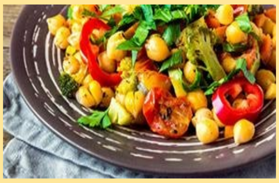

A good diet is essential to maintain health.
1. Try to add good fats to your diet like avocado, nuts, tuna, salmon and olive oil. They are good for your brain and heart.
2. Avoid excess of salt, both salt itself and salt from industrial foods. Salt increases blood pressure and high blood pressure increases the risk of heart attack and stroke.
3. Eat lots of fiber (vegetables, grains, fruits and legumes). They will make you feel full and can even help to control your blood sugar and cholesterol.
4. Make a colorful dish! The more colorful, the greater the variety of healthy nutrients you will eat.

In the last month, how often in your daily routine…	**(1) Always**	**(2) Often**	**(3) Seldom**	**(4) Never**	**(999) I prefer do not answer**
**Q29. Do you drink 5 or more doses (men) or 4 or more doses (women) of alcoholic beverages* on a single occasion, which means within 2 hours?**					
**Q30. Do you smoke tobacco (cigarette, electronic cigarette, cigar, pipe, smokeless tobacco)?**					
**Q31. Do you use marijuana or hashish?**					
**Q32. Do you use other drugs (cocaine, crack, amphetamines, ecstasy, opioids without medical prescription, and others)?**					

*1 dose of alcohol=1 glass of beer OR 1 glass of wine OR 1 shot of spirit (such as rum, vodka, whisky, tequila or gin).

Q33. Have you changed your substance use habits during the COVID-19 pandemic?
(1) Completely (2) Moderately (3) Slightly (4) Not at all (999) I prefer do not answer

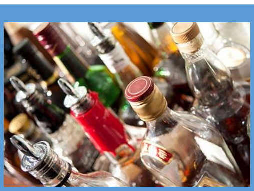

Do not smoke, do not drink alcohol, and do not use other drugs to deal with your emotions.

If you feel overwhelmed, talk to a health professional.

In the last month, how often in your daily routine…	(1) Always	(2) Often	(3) Seldom	(4) Never	(999) I prefer do not answer
**Q34.Do you exercise for at least 30 minutes daily (or 150 minutes a week)?**					
**Q35. Do you play at least 2 hours of team sports (like soccer, volleyball, basketball, rugby, etc.) a week?**					
**Q36. Do you choose to climb stairs instead of using an elevator and/or walking to perform your daily routines instead of using a car/public transportation?**					
**Q37. Do you feel good after performing physical activities?**					

Q38. Have you changed your physical activity routine during the COVID-19 pandemic?
(1) Completely (2) Moderately (3)Slightly (4) Not at all (999) I prefer do not answer

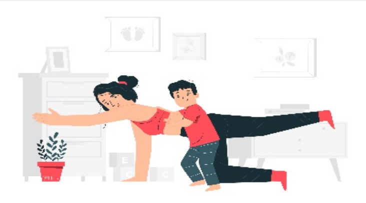

Regular physical activity is one of the most important things people can do to improve their health. Moving more and sitting less have tremendous benefits for everyone, improving overall health.
Some health benefits start immediately after activity, and even short bouts of physical activity are beneficial.

Try to keep physically active during the pandemic and self-isolation!

In the last month, how often in your daily routine…	(1) Always	(2) Often	(3) Seldom	(4) Never	(999) I prefer do not answer
**Q39. Do you make time to relax?**					
**Q40. Do you use any strategy or psychological support to deal with stress (for instance meditation, mindfulness or psychotherapy)?**					
**Q41. Do you use physical strategies to deal with stress (for instance yoga, tai-chi, exercise)?**					
**Q42. Do you practice a faith or religion?**					
**Q43.Do you feel that you have a good work-life balance?**					
**Q44. Do you feel that your work / chores are never done?**					
**Q45. Are you satisfied with the time it takes you to commute to work?**					
**Q46. Do you feel that your life has a meaning?**					
**Q47. Do you feel grateful for the life you have?**					

Q48. Have you changed your strategies to deal with stress during the COVID-19 pandemic?
(1) Completely (2) Moderately (3) Slightly (4) Not at all (999) I prefer do not answer

Taking care of yourself, your friends, and your family can help you cope with stress. Helping others cope with their stress can also make your community stronger.- Use strategies that helped you to deal with stress in the past.
- Take deep breaths, stretch, or meditate.
- Make time to unwind. Try to do some other activities you enjoy.
- Take breaks from watching, reading, or listening to the news, including social media. Hearing about the pandemic repeatedly can be upsetting.

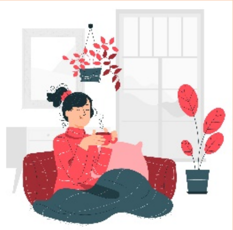

In the last month, how often in your daily routine…	(1) Always	(2) Often	(3) Seldom	(4) Never	(999) I prefer do not answer
**Q49. Do you manage to sleep between 7 and 9 hours per night?**					
**Q50. Do you feel rested with the number of hours you sleep?**					
**Q51. Do you usually rest (sleep or take a nap) after lunch?**					
**Q52. Do you maintain a regular sleep schedule?**					
**Q53. Do you use sleeping pills?**					

Q54. Have you changed your sleep pattern during the COVID-19 pandemics?
(1) Completely (2) Moderately (3) Slightly (4) Not at all (999) I prefer do not answer

Tips for Better Sleep

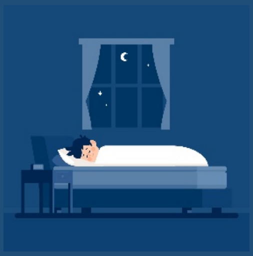

- **Be consistent. Go to bed at the same time each night and get up at the same time each morning, including on weekends**
- **Make sure your bedroom is quiet, dark, relaxing, and at a comfortable temperature**
- Remove electronic devices, such as TVs, computers, and smartphones, from your bedroom
- Avoid large meals, caffeine, and alcohol before bedtime
- Get some exercise. Being physically active during the day can help you fall asleep more easily at night.

**In the last month, how often in your daily routine…**	(1) Always	(2) Often	(3) Seldom	(4) Never	(999) I prefer do not answer
**Q55. Do you interact with your friends and/or relatives?**					
**Q56. Do you feel that you are part of a group of friends, the community or the society?**					
**Q57. Do you have someone you trust who listens to your problems**					

or concerns?					
Q58. Do you have someone to help with everyday chores (for instance cooking, housekeeping, shopping)?					
Q59. Do you have someone in your life to go out or have fun with when you feel like it?					
Q60. Do you take part in celebrations/ reunions with family/ friends/colleagues?					
Q61. Do you enjoy your leisure time?					
Q62. Do you make yourself available to support your significant ones?					
Q63. Are you satisfied with your sexual life?					
Q64. Do you feel loved?					

Q65. Have you experienced any changes regarding your social support during the COVID-19 pandemic?
(1) Completely (2) Moderately (3) Slightly (4) Not at all (999) I prefer do not answer

Connect with others

- Share your concerns and how you are feeling with a friend or a relative.
- Maintain healthy relationships, and build a strong support system.
- In case you live alone, try to stay connected with other people.

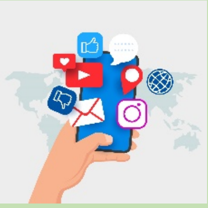

In the last month, how often in your daily routine…	(1) Always	(2) Often	(3) Seldom	(4) Never	(999) I prefer do not answer
Q66. Do you spend more than 2 hours a day watching TV, playing computer games, video games or in the Internet?					
Q67. Do you spend time on a computer / smartphone within one hour of going to sleep?					
Q68. Are you in touch with nature (for instance parks, beach, countryside, mountains)?					
Q69. Do you feel your relationship with nature, that is all living things, is an important part of who you are?					

Q70. Have you changed your pattern of indoor/outdoor time during the COVID-19 pandemic?
(1) Completely (2) Moderately (3) Slightly (4) Not at all (999) I prefer do not answer

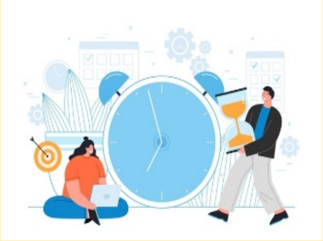

Tips:
- Try to reduce the amount of time you spend connected (screen time).
- Avoid using digital technology before bedtime. That causes a delay in falling asleep.
- Whenever possible, try to have at least 10 minutes of sunlight per day. This way you will help your body synthesize vitamin D, which is important for your overall health.

Q71. How would you rate your health in general?
(1) Very good (2) Good (3) Regular (4) Bad (5) Very bad (999) I prefer do not answer

**In the last 12 months** have you been diagnosed by a physician or health professional, OR have you received treatment for any of those following conditions?	No (0)	Diagnosis or treatment (1)	999. I prefer do not answer
Q72. Diabetes			
Q73. Heart disease			
Q74. Hypertension			
Q75.Anemia			
Q76.Asthma / bronchitis			
Q77.Depression			
Q78. Anxiety			
Q79. Schizophrenia			
Q80. Bipolar Disorder			
Q81. Anorexia /bulimia			
Q82. HIV/AIDS			
Q83. Cancer			
Q84. Tuberculosis			
Q85. Cirrhosis			
Q86. Kidney disease			
Q87. Dengue			
Q88. Chikungunya			
Q89. Others			

**Over the last 2 weeks**, how often have you been bothered by any of the following problems?	(0) Not at all	(1) Several	(2) More than half the days	(3) Nearly every day	(999) I prefer do not answer
Q90. Little interest or pleasure in doing things.					
Q91. Feeling down, depressed, or hopeless.					
Q92. Feeling nervous, anxious, or on edge.<br>Q93. Not being able to stop or control worrying.					
Q94. Worrying too much about different things.					
Q95. Trouble relaxing.					
Q96. Being so restless that it's hard to sit still.					
Q97. Becoming easily annoyed or irritable.					
Q98. Feeling afraid as if something awful might happen.					

99. How often do you have a drink containing alcohol?
(0) Never
(1) Monthly or less
(2) 2-4 times a month
(3) 2-3 times a week
(4) 4 or more times a week

999. I prefer do not answer

100. How many standard drinks containing alcohol do you have on a typical day?
(0) 1 or 2
(1) 3 or 4
(2) 5 or 6
(3) 7 to 9
(4) 10 or more
999. I prefer do not answer or not applicable

101. How often do you have six or more drinks on one occasion?
(0) Never
(1) Less than monthly
(2) Monthly
(3) Weekly
(4) Daily or almost daily
999. I prefer do not answer

It is normal feeling sad, scared, stressed, confused, or annoyed during a crisis. Talking with people you trust can help. Keep in touch with your friends and family.

Have a plan: Where should I go and how I will look for help in any physical or mental needs, if it would be necessary?

FINAL SCREEN

Thank you very much for participating, your answers are very important!

Let´s all maintain hygiene habits and follow the recommendations of the World Health Organization and local authorities. This is the best way to help reduce transmission.

If you have questions about COVID-19, search and share only reliable and up-to-date information on websites of institutions such as the World Health Organization (https://www.who.int/emergencies/diseases/novel-coronavirus-2019) the ministry of health (https://coronavirus.saude.gov.br), and Fiocruz (https://portal.fiocruz.br/coronavirus).
