# Supplementary material for: Depression, Anxiety, and Lifestyle Among Essential Workers: A Web Survey From Brazil and Spain During the COVID-19 Pandemic
Source: J Med Internet Res. 2020 Oct 30;22(10):e22835. doi: 10.2196/22835 (PMC7641648; doi:10.2196/22835)
Supplement: Multimedia Appendix 2 [file jmir_v22i10e22835_app2.docx]

**Supplementary material 2**

1. **Statistical analysis**

**(1) Dealing with non-**r**esponses**

A straightforward imputation procedure using median values was used in order to deal with non-response (i.e. the option: “I prefer not to answer it” in the online questionnaire). The median was found to be the best measure of central tendency for the sake of getting an optimal consistency of the items belonging to the different scales. Each item of PHQ-2, GAD-7, SMILE-C and AUDIT-C scales was assessed for its distribution, measures of central tendency, and dispersion (stratified by country). The overall scores and the frequencies of positive screenings were compared before and after imputation to evaluate possible changes. Irrespective of the item or country, the proportion of imputed values never exceed 1.0%.

**(2) Variable selection**

In every case there is a large number of independent variables to be putatively entered into a multinomial logistic regression model, some methods and techniques may be used in order to find the most parsimonious set of variables. Such procedures aim to avert overfitting and the choice of a model to be putatively found − after the proper model diagnosis − to be less than optimal in terms of its goodness of fit.

We chose to use LASSO (*Least Absolute Shrinkage and Selection Operator*), as one of the possible strategies under the conceptual umbrella of the different “*shrinkage methods*”, as well as an extension of the minimum square regression. The latter aims to minimize the residuals of the sum of the squared differences between expected and observed values (Musoro et al., 2014).

The LASSO method adds a penalty term to the estimation function of *β_i_* coefficients. LASSO profits from the so-called L1 regularization, by the way of adding a λ penalty to the sum of absolute values of the coefficients.

According to Zou and Hastie (2005), in the context of LASSO variables found to be less relevant are forced to assume null (zero) values. Assuming null values, such variables can no longer contribute to further reduce the mean quadratic error.

The minimum square minimization of errors of the *β_i_* coefficients can be described by equation 1 as follows:

| 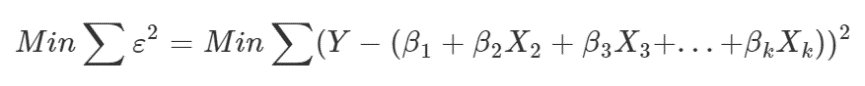 | (1) |
| --- | --- |

In the context of LASSO, the λ penalty is included in equation 1 as follows:

| 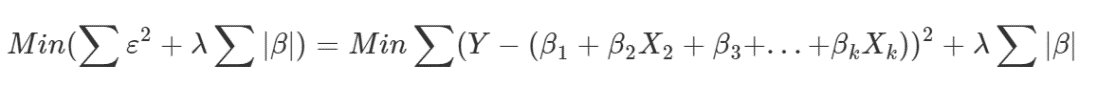 | (2) |
| --- | --- |

The λ penalty assumes, in every single case, a positive value. The choice of an optimal value for λ must be found after an iterative process departing from the definition of an a priori range to be repeatedly tested until obtaining the best estimates.

LASSO was implemented based on R 4.0.2 libraries as follows: glmnet (Lasso and Elastic-Net Regularized Generalized Linear Models)

Besides LASSO, the subset of variables corresponding to the model with the best goodness of fit, as defined after the best R^2^ correlation coefficients using R library and respective function *regsubsets* (Function for Model Selection - Generic function for regression subset selection with methods for formula and matrix arguments).

Only variables with a p-value <0.20 in the chi-square test for association were entered into the multinomial logistic regression models.

**(2.1) Graphic presentation of the preliminary findings respecting variable selection**:

**(2.1.1) Regsubsets Function:**


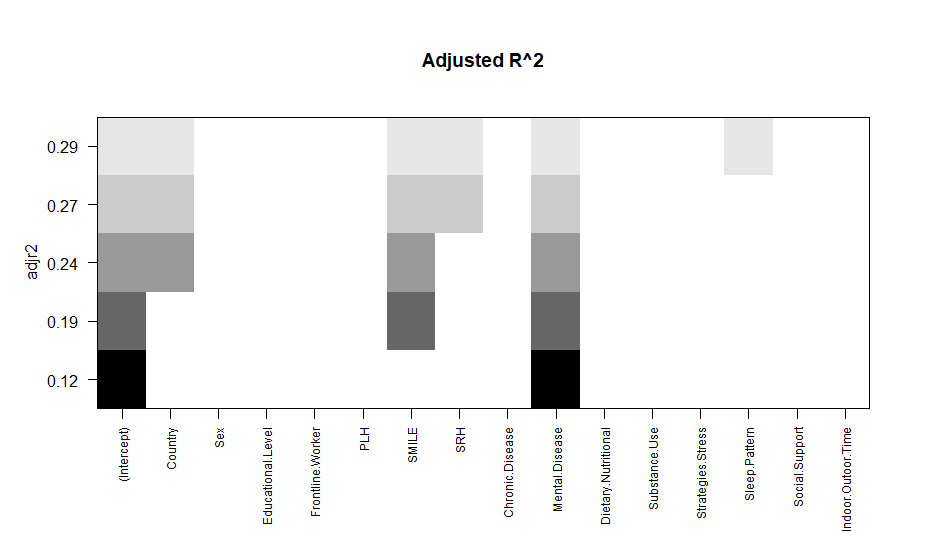


Notes: PLH: People Living in the House; SMILE: Short Multidimensional Inventory Lifestyle Evaluation: Confinement; SRH: Self-Reported Health

**(2.1.2) Lasso and Elastic-Net Regularized Generalized Linear Models**

Model summary output

**print(fit)**

Call: glmnet(x = x, y = y, family = "multinomial", alpha = 1, type.multinomial = "grouped")

Df % Dev Lambda

1 0 0·00 0·212900

2 2 0·96 0·193900

3 2 2·30 0·176700

4 4 3·56 0·161000

5 4 5·10 0·146700

6 5 6·52 0·133700

7 5 7·79 0·121800

8 6 8·94 0·111000

9 6 10·02 0·101100

10 6 10·96 0·092140

11 6 11·76 0·083950

12 6 12·46 0·076500

13 6 13·07 0·069700

14 6 13·60 0·063510

15 6 14·05 0·057870

16 6 14·45 0·052730

17 7 14·81 0·048040

18 8 15·18 0·043770

19 9 15·51 0·039890

20 9 15·82 0·036340

21 9 16·08 0·033110

22 10 16·32 0·030170

23 11 16·53 0·027490

24 12 16·74 0·025050

25 14 16·95 0·022820

26 15 17·17 0·020800

27 15 17·37 0·018950

28 15 17·54 0·017270

29 16 17·69 0·015730

30 16 17·82 0·014330

31 16 17·94 0·013060

32 17 18·04 0·011900

33 17 18·14 0·010840

34 17 18·22 0·009880

35 17 18·29 0·009002

36 17 18·34 0·008202

37 17 18·39 0·007474

38 17 18·43 0·006810

39 17 18·47 0·006205

40 17 18·50 0·005654

41 17 18·52 0·005151

42 17 18·54 0·004694

43 17 18·56 0·004277

44 18 18·57 0·003897

45 18 18·59 0·003551

46 18 18·60 0·003235

47 18 18·61 0·002948

48 18 18·61 0·002686

49 18 18·62 0·002447

50 18 18·63 0·002230

51 18 18·63 0·002032

52 18 18·63 0·001851

53 18 18·64 0·001687

54 18 18·64 0·001537

55 18 18·64 0·001400

56 18 18·64 0·001276

57 18 18·65 0·001163

58 18 18·65 0·001059

59 18 18·65 0·000965

Minimum parameter for λ

**lasso.cv$lambda.min**

[1] 0·005653636

Parameter λ SE

**lasso.cv$lambda.1se**

[1] 0·01894874


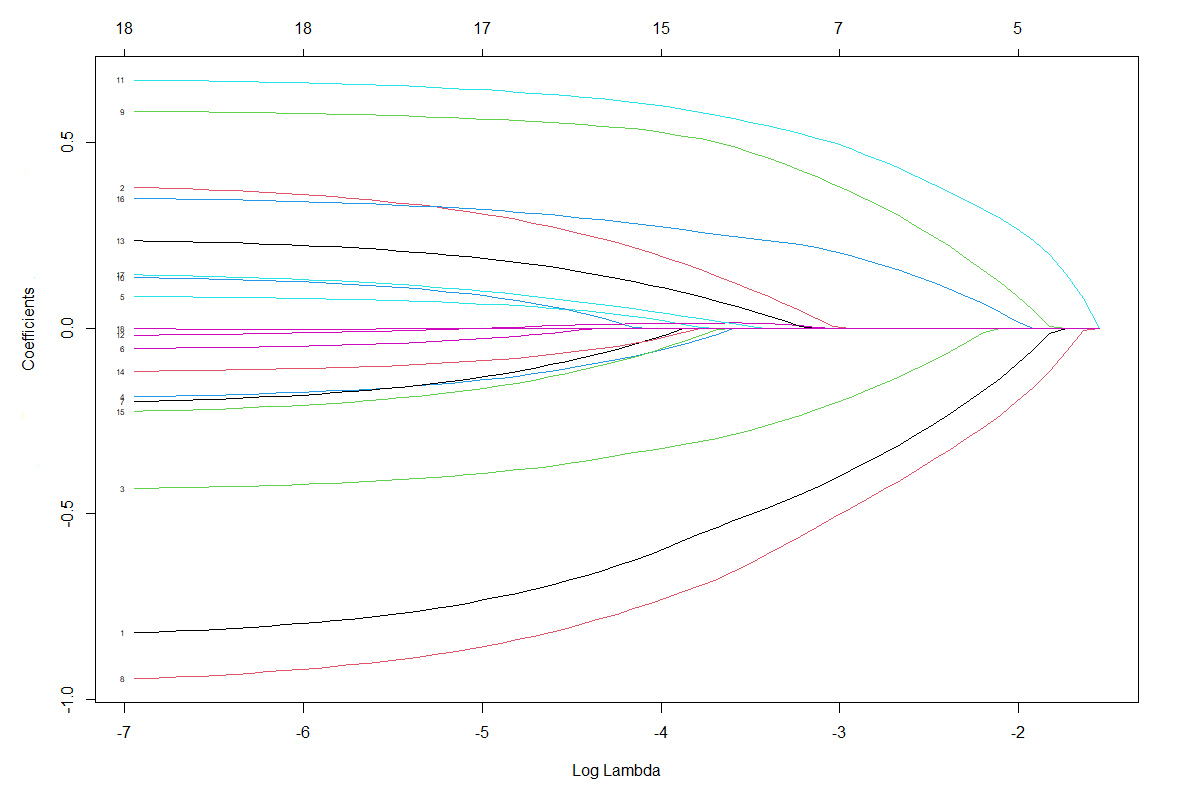


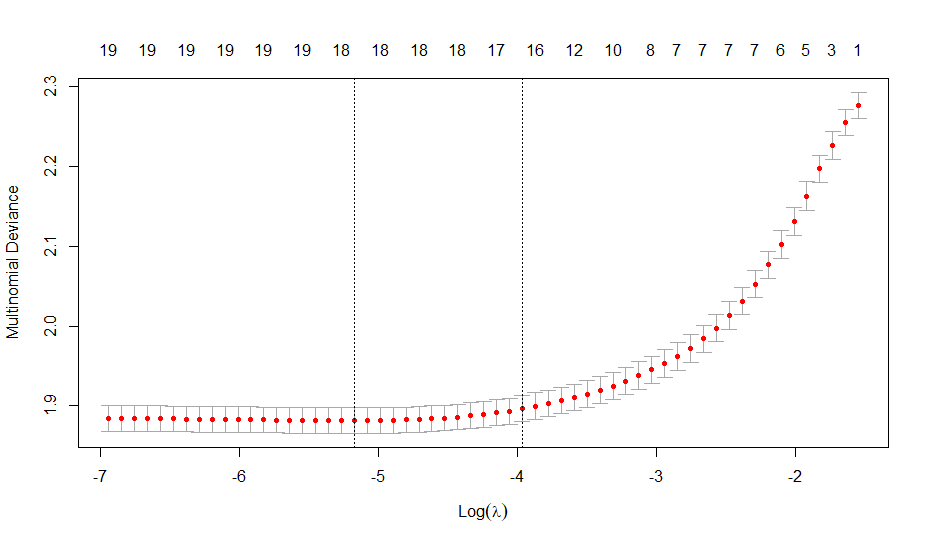


**(3) Final multinomial model**

The final model comprises the variables selected after the abovementioned steps (2.1) & (2.2) as follows:

**Table 2: Independent variables included in the final model**

| **Variables** | **Positive for depression only** | | **Positive for anxiety only** | | **Positive for both** | | |
| --- | --- | --- | --- | --- | --- | --- | --- |
|  | **Exp(IC95%)** | **p_value** | **Exp(IC95%)** | **p_value** | **Exp(IC95%)** | **p_value** |  |
| **Country** |  |  |  |  |  |  |  |
| Brazil | 2·89 (2·07-4·06) | 0·000 | 2·81 (2·11-3·74) | 0·000 | 5·99 (4·53-7·91) | 0·000 |  |
| Spain |  |  |  |  |  |  |  |
| **Sex** |  |  |  |  |  |  |  |
| Male |  |  |  |  |  |  |  |
| Female | 1·40 (1·06-1·85) | 0·018 | 1·38 (1·07-1·76) | 0·011 | 1·90 (1·53-2·36) | 0·000 |  |
| **Age** |  |  |  |  |  |  |  |
| Up to 41y old | 2·11 (1·63-2·73) | 0·000 | 1·49 (1·19-1·85) | 0·000 | 2·69 (2·21-3·26) | 0·000 |  |
| 42+ y old |  |  |  |  |  |  |  |
| **Frontline worker** |  |  |  |  |  |  |  |
| Yes | 0·81(0·60-1·08) | 0·149 | 1·49 (1·18-1·87) | 0·000 | 1·25 (1·02-1·53) | 0·029 |  |
| No |  |  |  |  |  |  |  |
| **SMILE-C (score)** |  |  |  |  |  |  |  |
| Up to 85 | 4·00 (2·72-5·87) | 0·000 | 2·39 (1·80-3·20) | 0·000 | 8·30 (5·90-11·7) | 0·000 |  |
| 86 or more |  |  |  |  |  |  |  |
| **Self-rated health** |  |  |  |  |  |  |  |
| Very good/good |  |  |  |  |  |  |  |
| Regular/bad/very bad | 1·81 (1·33-2·45) | 0·000 | 1·83 (1·40-2·40) | 0·000 | 3·69 (2·97-4·59) | 0·000 |  |
| **Diagnosed or treated for mental health diseases** |  |  |  |  |  |  |  |
| Yes | 1·40 (1·03-1·90) | 0·031 | 1·91 (1·47-2·47) | 0·000 | 3·52 (2·86-4·35) | 0·000 |  |
| No |  |  |  |  |  |  |  |
| **Sleep pattern changes** |  |  |  |  |  |  |  |
| No/mild |  |  |  |  |  |  |  |
| Moderate/complete | 1·58 (1·22-2·04) | 0·000 | 1·86 (1·48-2·32) | 0·000 | 2·29 (1·90-2·77) | 0·000 |  |

**Model Fitness**

*a.* Hosmer and Lemeshow goodness-of-fit test

Chi-Square = 14·734, df = 24, **p-value = 0·9283**

*b.* Likelihood Ratio Tests

Chi-Square = 1338·373, df = 24, **p-value= 0·000**

**Analysis of residuals (re: linearity assumption)**


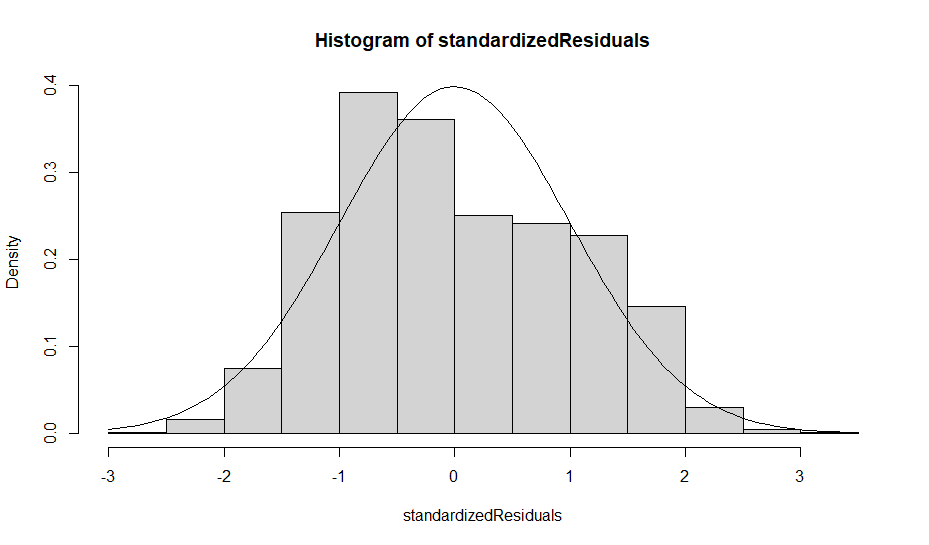


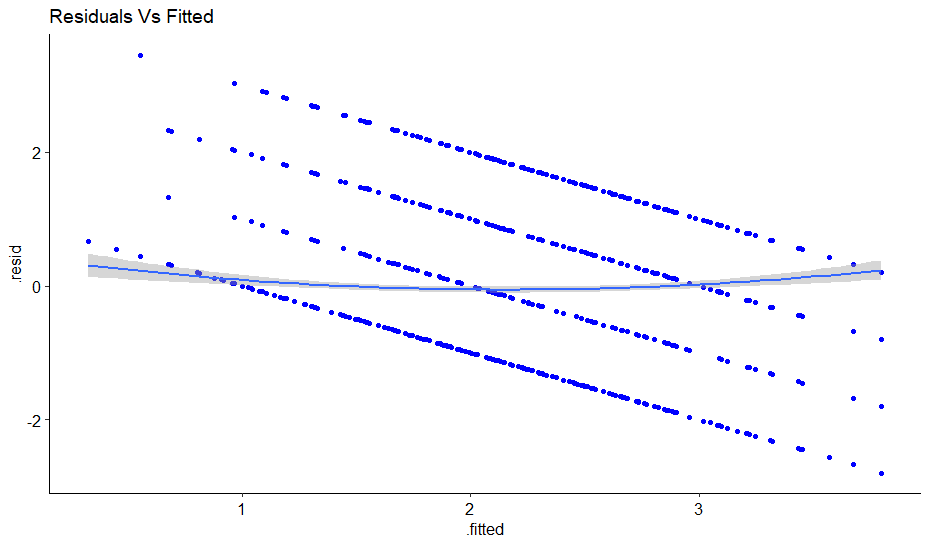


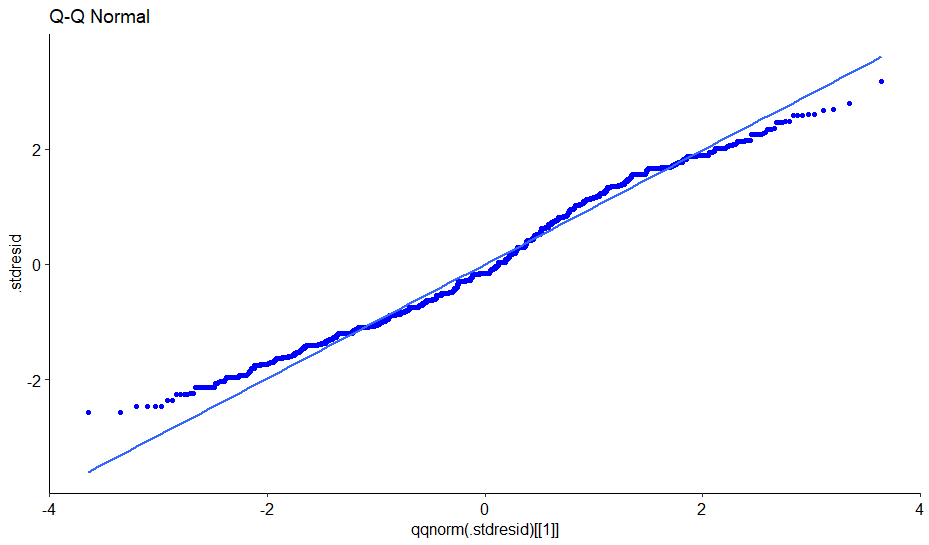


**References**:

de Jong VMT, Eijkemans MJC, van Calster B, et al. Sample size considerations and predictive performance of multinomial logistic prediction models. Stat Med 2019; 38:1601–1619. doi: 10.1002/sim.8063.

Smith GC, Seaman SR, Wood AM, Royston P, White IR. Correcting for optimistic prediction in small data sets. Am J Epidemiol 2014;180(3):318-324. doi:10.1093/aje/kwu140

Zou H, Hastie T. Regularization and variable selection via the elastic net. J. R. Statist. Soc. B 2005.67(2):301–320. doi: 10.1111/j.1467-9868.2005.00503.x

Tutz G, Pößnecker W, Uhlmann L. Variable selection in general multinomial logit models, Comput Stat Data Anal 2015. 82: 207-222. doi:10.1016/j.csda.2014.09.009.

Musoro JZ, Zwinderman AH, Puhan MA, ter Riet G, Geskus RB. Validation of prediction models based on lasso regression with multiply imputed data. BMC Med Res Methodol 2014. 14:116. doi:10.1186/1471-2288-14-116
